# Inflammatory cell-associated tumors. Not only macrophages (TAMs), fibroblasts (TAFs) and neutrophils (TANs) can infiltrate the tumor microenvironment. The unique role of tumor associated platelets (TAPs)

**DOI:** 10.1007/s00262-020-02758-7

**Published:** 2020-11-03

**Authors:** Violetta Dymicka-Piekarska, Olga M. Koper-Lenkiewicz, Justyna Zińczuk, Ewa Kratz, Joanna Kamińska

**Affiliations:** 1 Department of Clinical Laboratory Diagnostics, Medical University of Bialystok, Waszyngtona 15A, 15-269 Bialystok, Poland; 2 Department of Laboratory Diagnostics, Faculty of Pharmacy, Wroclaw Medical University, Borowska Street 211A, 50-556 Wrocław, Poland

**Keywords:** Tumor associated platelets (TAPs), Platelet activation, Cancer development, Tumor microenvironment

## Abstract

It is well known that various inflammatory cells infiltrate cancer cells. Next to TAMs (tumor-associated macrophages), TAFs (tumor-associated fibroblasts) and TANs (tumor-associated neutrophils) also platelets form the tumor microenvironment. Taking into account the role of platelets in the development of cancer, we have decided to introduce a new term: tumor associated platelets—TAPs. To the best of our knowledge, thus far this terminology has not been employed by anyone. Platelets are the first to appear at the site of the inflammatory process that accompanies cancer development. Within the first few hours from the start of the colonization of cancer cells platelet-tumor aggregates are responsible for neutrophils recruitment, and further release a number of factors associated with tumor growth, metastasis and neoangiogenesis. On the other hand, it also has been indicated that factors delivered from platelets can induce a cytotoxic effect on the proliferating neoplastic cells, and even enhance apoptosis. Undoubtedly, TAPs’ role seems to be more complex when compared to tumor associated neutrophils and macrophages, which do not allow for their division into TAP P1 and TAP P2, as in the case of TANs and TAMs. In this review we discuss the role of TAPs as an important element of tumor invasiveness and as a potentially new therapeutic target to prevent cancer development. Nevertheless, better exploring the interactions between platelets and tumor cells could help in the formulation of new therapeutic goals that support or improve the effectiveness of cancer treatment.

**Electronic supplementary material:**

The online version of this article (10.1007/s00262-020-02758-7) contains supplementary material, which is available to authorized users.

## Background/Introduction

The association between inflammation and cancer has long been the subject of numerous studies. In 1863, Virchow put forward a hypothesis stating that immune cell infiltrations reflect the site of neoplastic lesions within the chronically affected tissue [1]. A decade later, Dvorak reported that carcinogenesis and inflammatory condition share some growth mechanisms, such as cell proliferation, increased survival, migration and enhanced angiogenesis, which are strictly controlled by growth factors, proinflammatory cytokines and proangiogenic factors. Moreover, he observed that cells involved in inflammation infiltrate the neoplastic tissue as well [2, 3].

Transforming neoplastic cells are surrounded by numerous cells: fibroblasts, endothelial cells, pericytes and mesenchymal cells that together form tumor stroma. These cells produce a number of cytokines and chemokines, which attract the subsequent populations of immune cells, including macrophages, neutrophils, mast cells, dendrites and T, B and NK cells which by infiltrating tumor tissues form the tumor microenvironment [4]. Leukocytes may account for up to 50% of total tumor mass. The interaction of cancer cells with macrophages activates them to produce cytokines, e.g. IL-8 which stimulates further inflow of inflammatory cells [5, 6]. In the tumor microenvironment, cells communicate with one another via direct contact or production of various mediators [7]. The activity of inflammatory cells and the type and level of the expression of factors that modulate inflammation affects the balance between their pro- and antineoplastic activity. In developed tumors, the inflammatory cells act in favor of the tumor, increasing the survival and proliferation of the transformed cells [8, 9].

### Tumor-associated macrophages (TAMs)

Macrophages are the main cells playing a role in the inflammatory condition accompanying neoplastic disease. They are derived from precursor cells-monocytes, activated by MCP-1/CCL2 (monocyte chemoattractant protein-1), cytokines (IL-6, CSF-1, VEGF) and chemokines (CCL2, CCL5, CCL8) produced by neoplastic cells. Macrophages are diverted from blood circulation to tissues where they differentiate from mature forms [10]. Present in the tumor microenvironment they are defined as tumor associated macrophages (TAMs). TAMs can promote tumor cell death and preclude the formation of new blood vessels. At the same time they produce proangiogenic factors, growth factors and extracellular matrix metalloproteinases, which in turn may stimulate cancer growth [11, 12].

The opposite action of these cells is associated with the presence of two TAM phenotypes, namely M1 and M2 [12] (Suppl. Fig. 1). M1 macrophages are stimulated by IFN-γ, LPS, TNF-α or GM-CSF, and inhibit tumor growth by producing pro-inflammatory cytokines such as: IL-1, -6, -8, -12, -23, TNF-α, small quantities of IL-10 as well as ROS (reactive oxygen species) and RNI (reactive nitrogen intermediates) [13]. These cells affect Th1 activation and enhance antineoplastic response. They can recognize tumors and kill tumor cells by cytotoxic effect (e.g. ROS) [14]. On the contrary, M2 macrophages display pro-tumor activity. M2, activated by IL-4 and -13, produce small amounts of IL-12 and -23 and large quantities of IL-10, which silences an acute inflammatory state and attenuates antineoplastic immune response of the body [8, 12, 15]. M2 macrophages have an effect on the inactivation of *T* cells, which crucially decreases the body’s ability to resist cancer development and progression [16]. TAMs can also promote tumor angiogenesis and metastatic potential by the synthesis of cytokines such as: IL-6, -17, -23, and inhibit cytotoxic *T* cell responses [11].

Due to varying locations, certain TAMs produce growth factors (EGF, PDGF, IL-6, IL-8), or generate immunosuppressive factors (IL-10, TGF-β, PGE2) [12], whereas others produce metalloproteinases (MMP7, MMP9, MMP12), urokinase plasminogen activator (uPA) and cathepsin B able to transform the extracellular matrix (ECM) [17]. In this way, these cells promote tumor proliferation and growth, tissue repair and remodeling and angio- and lymphangiogenesis through the production of VEGF, TNF-α and IL-8, and exert an immunosuppressive effect on the inflammatory cells [18]. The tumor microenvironment is formed first of all by TAM M2. A growing tumor uses substantial amounts of nutrients and oxygen, but lack of contact with the network of blood vessels inside it frequently leads to hypoxia. Hence, angiogenesis is an indispensable stage of cancer progression. The development of new blood vessels depends on the recruitment of TAMs which recognize hypoxia-associated signals. The production and release of TAMs as well as other cells of important proangiogenic factors, such as VEGF, IL-8 (CXCL8), EGF, CXCL1 and hypoxia- induced factor 1 alpha (HIF1α) are regulated by NF-*k*B, STAT3 and AP-1 [19]. Under the hypoxia condition, HIF-1α stimulates the expression of CXCL12, which leads to the activation and recruitment of endothelial cells [12]. Hypoxia decreases the expression of MMP-9 and TIMP-1 by increasing the expression of disintegrin and metalloproteinase domain-containing protein 8 & 9 coding genes (*ADAM8* & *ADAM9*) [16]. HIF-1α is also the major transcription factor affecting the synthesis of VEGF—the major factor in the initial stages of new blood vessels [12].

TAMs, especially TAM M2, have a significant potential as diagnostic biomarkers in many cancers: breast, gastric, prostate and pancreatic cancer [16]. According to the newest knowledge TAMs could also be used as a prognostic biomarker in lung, esophageal squamous cell and bladder cancer [20].

### Tumor-associated fibroblasts (TAFs)

During tumor development, its stromal fibroblasts also undergo some phenotypic changes [21]. Tumor associated fibroblasts (TAFs) do not have a direct effect on cancer growth but they affect healthy fibroblasts in the tumor microenvironment, changing their phenotype to the proneoplastic one. The origin and role of TAFs are poorly understood but TAFs in the tumor environment become activated and secrete a multitude of factors involved in tumorigenesis. TAFs produce extracellular matrix proteins (collagen and fibronectin), basement membrane proteins and metalloproteinases (MMP-1, -7, -9). Other soluble factors secreted by TAFs: IL-10, -13 possess an immunosuppressive effect, and inhibit synthesis of pro-inflammatory cytokines (e.g. IFN-γ, TNF-α, IL-2, -3) and TGF-β, can promote cancer invasiveness and metastasis [22]. TAFs can also promote neoangiogenesis by synthesis of VEGF [12].

### Tumor-associated neutrophils (TANs)

Neoplastic cells producing chemokines (e.g. CXCL1), cytokines (TNF-α and IFN-γ) and adhesion proteins can also recruit neutrophilic granulocytes to the tumor microenvironment. Tumor associated neutrophils (TANs) have both pro- and anti-neoplastic functions. Similarly to macrophages, they have two phenotypes: N1 can inhibit tumor growth by increasing the expression of MMP-8, inducing apoptosis via FAS pathway activation and as a result of triggering antibody-dependent cellular cytotoxicity [23]. The N2 phenotype shows a proneoplastic action through the enhancement of MMP-9 expression and release of CXCL-1 and -6, which recruit new proinflammatory cells to the tumor microenvironment. As a consequence of tumor growth and derived cytokines (IL-8 and G-CSF), also NETs (neutrophil extracellular traps) are formed, possibly as a side-effect of the cytokine environment [24]. Its role in cancer is still unclear and the first description of tumor-induced NET formation was published in 2012 [25]. Formation of NETs may seem to be beneficial in fighting infection, as more NETs have a prothrombotic effect inducing intrinsic coagulation pathway, by binding and activation factor XII [26]. It seems that NETs in tumor-bearing mice were associated with the formation of thrombi in the lungs. Experimental research in cancer-bearing mice indicates that NETs also accumulate in the peripheral circulation, cause systemic inflammation and significantly reduce vascular function in the organs that are not sites for either primary or metastatic tumor growth [27].

### Tumor-associated platelets (TAPs)

However the phrase “tumor associated platelets” can be found in an earlier publication [28], we are the first to postulate that this term and its abbreviation (TAPs) should be commonly used in the scientific literature, along with TAMs, TAFs, and TANs. Taking into account the role of platelets in the development of cancer, it is justified to introduce the term TAPs. To the best of our knowledge, so far nobody has used this abbreviation, but its use seems to be justified. However, it is difficult to make such a strict division into TAP P1 and P2 as in the case of neutrophils and macrophages, because TAPs’ role is undoubtedly more complex. One example might be the role of CD40 ligand (CD40L) secreted from the granules of active platelets [29]. On the one hand CD40L can induce apoptosis of cancer cells, while on the other hand it can enhance tumor growth and progression [30]. The effect induced by CD40L appears to depend not only on the type of cells that show the receptors’ expression but also on the strength of the signal transmitted by the ligand. The strong signal (high number of CD molecules)—induces apoptosis of cancer cells, whereas the weak signal (small number of CD receptors for the ligand) promotes tumor growth [31].

Tumor-associated platelets (TAPs) like other cells infiltrate the tumor environment, although reports on the effect of blood platelets on tumor growth are inexplicit. It has been indicated that factors released from platelets can induce a cytotoxic effect on the proliferating neoplastic cells, cause mitotic arrest in the G0/G1 phase, and even enhance apoptosis. However, many researchers seem to suggest an active role for platelets in tumor growth and metastasis formation [32].

**Fig. 1:**
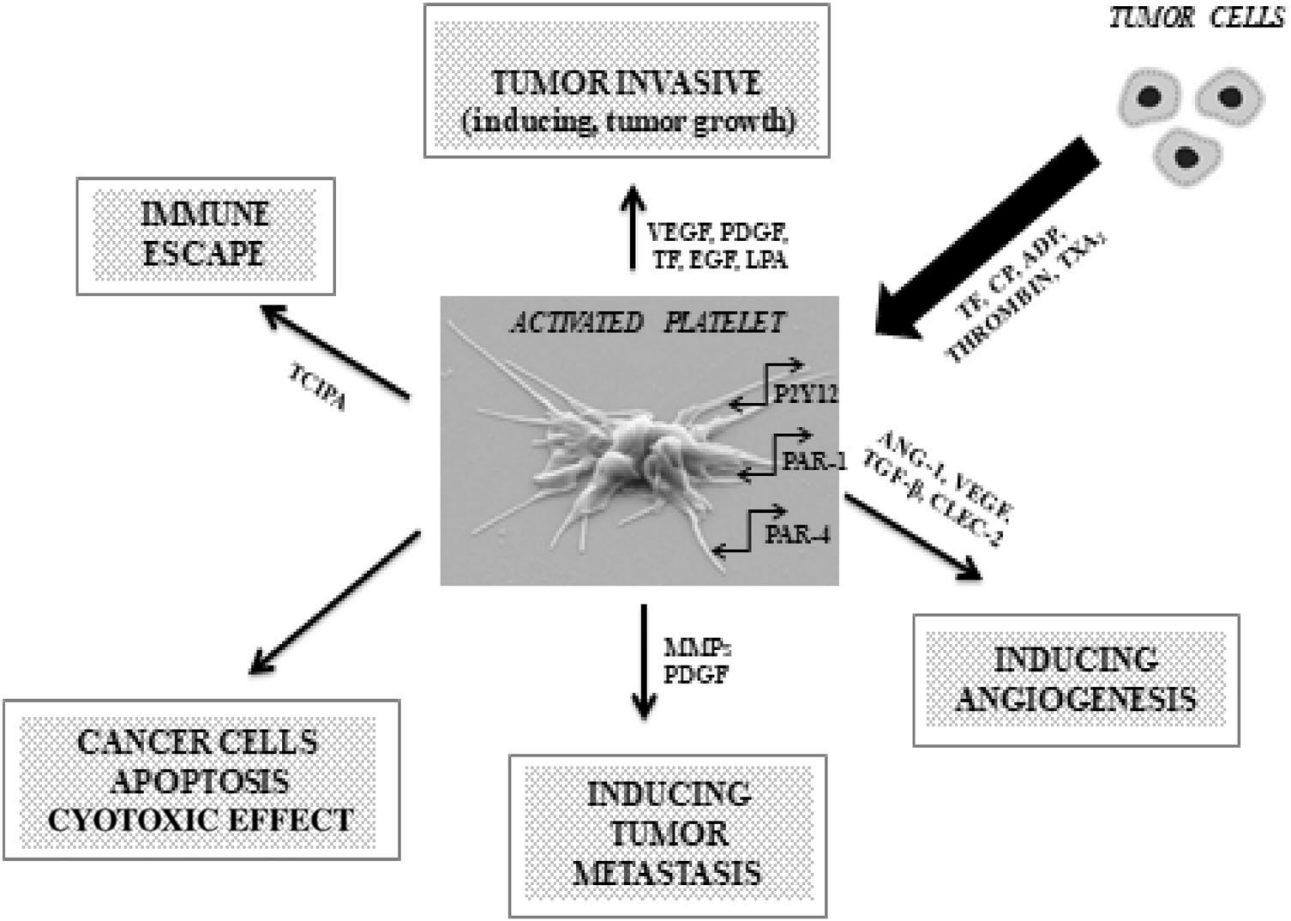
Platelet activity in cancer. *ADP* adenosine diphosphate, *ANG-1* angiopoietin 1, *CLEC-2* C-type lectin receptor 2, *CP* cancer procoagulant, *EGF* epidermal growth factor, *LPA* lysophosphatidic acid, *MMPs* matrix metalloproteinases, *P2Y12* chemoreceptor for adenosine diphosphate, *PAR -1, -4* protease-activated receptor -1, -4, *PDGF* platelet-derived growth factor, *TCIPA* tumor cell-induced platelet aggregation, *TF* tissue factor, *TGF-β* transforming growth factor β, *TXA*_*2*_ Thromboxane A_2_, *VEGF* vascular endothelial growth factor

The interactions of blood platelets with cancer cells are complex. The latter induce platelet activation and aggregation both directly and indirectly. Cancer-dependent platelet activation involves both substances secreted by tumor cells and surface molecules of tumor cells [33] (Fig. 1).

**Fig. 2:**
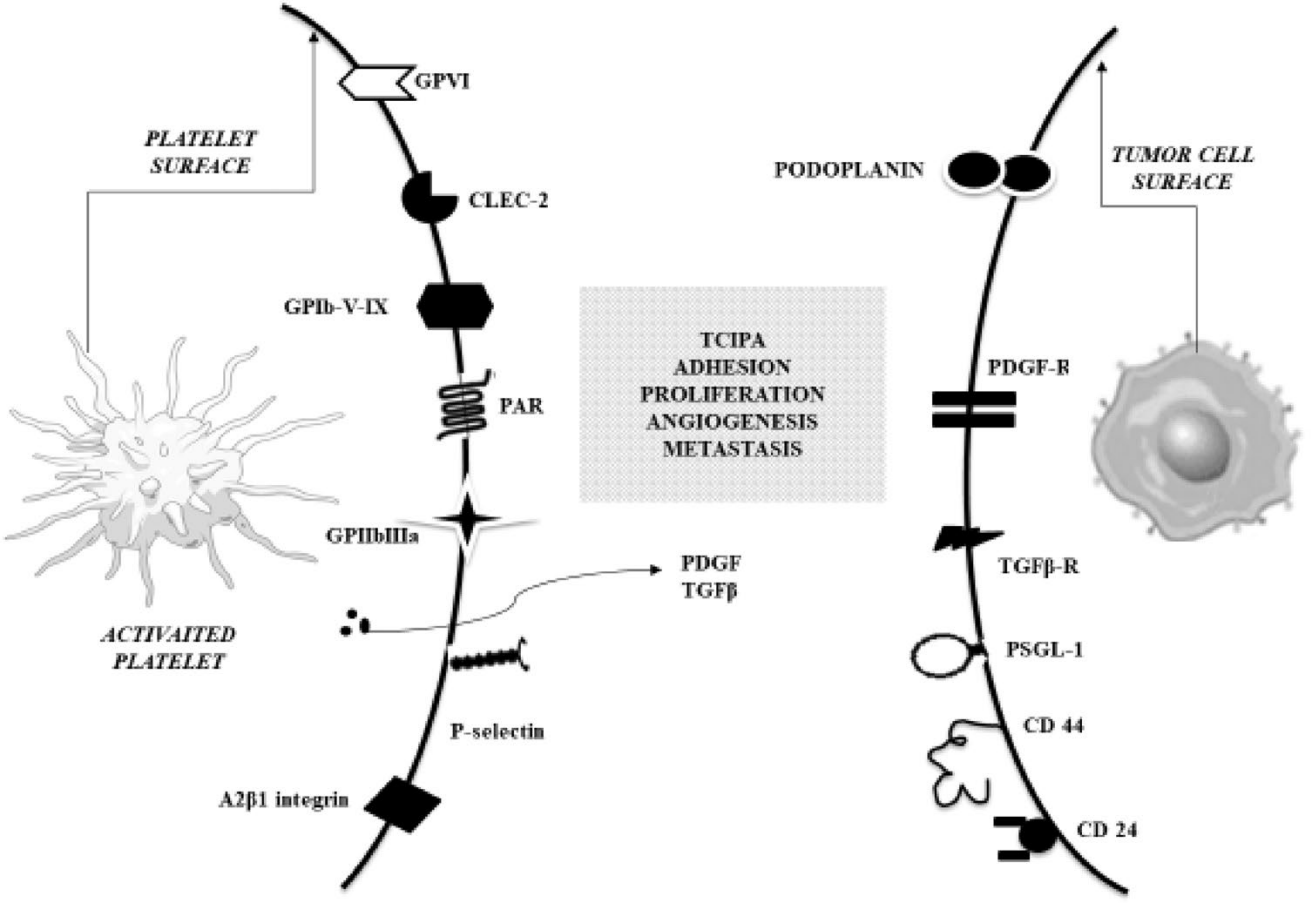
Binding of platelet receptors to ligands on cancer cells. *CD* cluster of differentiation, *CLEC-2* C-type lectin receptor 2, *GPIb-V-IX* glycoprotein Ib-V-IX complex, *GPIIbIIIa* glycoprotein IIb/IIIa, integrin αIIbβ3, *GPVI* glycoprotein VI, *PAR* protease-activated receptor, *PDGF* platelet-derived growth factor, *PDGF-R* platelet-derived growth factor receptor, *PSGL-1* P-selectin glycoprotein ligand-1, *TCIPA* tumour cell-induced platelet aggregation, *TGF-β* transforming growth factor β, *TGF-β-R* transforming growth factor β receptor, *α2β1 integrin* also known as VLA-2, GPIa-IIa, CD49b

Tumor cells-platelet binding is possible due to platelet receptors and molecules present on the platelet surface, e.g. GP Ib-IX-V, GP IIb-IIIa, GP V, P-selectin and CLEC-2 (C-type lectin-like receptor 2) (Fig. 2). Newly discovered CLEC-2 and its activator, podoplanin (PDPN) – expressed on multiple tumor cells (e.g. colorectal, lung and bladder carcinomas), are key in platelet aggregation and seem to be important in platelet-cancer cell interaction [34][35][36]. It is possible that CLEC-2 inhibitors could be the new potential antitumor therapeutic target. In an experimental study with several monoclonal antibodies against PDPN-CLEC-2 interactions revealed a decrease in the hematogenous metastasis rate without significantly increasing risk of bleeding [37][38].

Platelets are indirectly activated by tumor cells via coagulation activation induced mainly by TF present on tumor cells [39]. Thus, on the one hand platelets can affect the development of the tumor microenvironment, vascular neoangiogenesis and as a consequence metastasis formation. On the other hand, however, cancer cells themselves stimulate blood platelets by secreting TF, CP and collagen, and by using them to initiate processes that warrant survival and proliferation [40]. Adhesion and tumor cell-induced platelet aggregation are very important processes contributing to cancer progression regulated by platelets and their granular contents. Platelet aggregation in response to tumor cell stimulation is known as tumor cell-induced platelet aggregation (TCIPA) [41]. Blood platelets are also a source of TGF-β (transforming growth factor-β), which as an activator of the NF-κB signaling pathway in transformed cells stimulates cancer progression e.g. by promoting metastasis formation [42]. It also stimulates differentiation of endothelial cells into mesenchymal cells able to migrate and proliferate. The epithelial-mesenchymal transition (EMT) is an indispensable process promoting cancer metastases [38]. Platelet molecules such as P-selectin and GP IIb/IIIa have also been implicated in platelet-induced NET formation [40][27]. It is believed that NETs constitute a scaffold for platelet activation and thrombus formation.

### Platelets in repressing immune response

To survive in the circulatory system, tumor cells need to finagle immune cells. Therefore they turn to subvert the immune system by hijacking its antitumor properties or attracting immunosuppressive cells. For example, tumor cells may bear a resemblance to platelets by presenting several platelets’ receptors [28]. Circulating tumor cells can also bind to platelets, which protects them from NK cells. This is achieved by the translocation of the major histocompatibility complex class I (MHC class I) from platelets to the tumor cells, resulting in the antitumor properties of NK cells. Moreover, platelets release TGF-β, which reduces the expression of natural-killer group 2, member D on NK cells (NKG2D), and thus leads to the impairment of interferon-γ production and NK cell cytotoxicity. TGF-β also inhibits NK cells activation/function via the suppression of mTOR activity [28, 43, 44].

Platelets can also exert antitumor immunity activity through the debilitation of tumor-infiltrating lymphocytes (TILs). TGF-β released from platelets converts CD4 + (Tcony) cells into induced regulatory *T* (iTreg) cells, which kills activated *T* cells in a granzyme B (GzmB)-dependent fashion [44]. Moreover, platelets constitutively express the non-signaling TGF-β-docking receptor glycoprotein A repetitions predominant (GARP) [28]. The role of GARP relies on increases of the functional TGFβ activation in the neighborhood of GARP-expressing cells, which encompass regulatory *T* (Treg) cells. Rachidi et al. [45] show that the major source of TGFβ in the circulatory system and in the tumor microenvironment are platelets. This is achieved by a constitutive expression of GARP rather than secretion of TGFβ per se. GARP-TGFβ complex on platelets can be formed intracellularly, via the de novo biogenesis, or extracellularly where latent TGFβ (LTGFβ) is snatched by and bound to GARP in the extracellular matrix from non-platelet sources [45]. Besides TGFβ, also other soluble factors released from platelets, mostly lactate, may suppress: *T* cell proliferation, blastogenesis, and IFNγ production [44, 45].

The discovery of platelet-mediated *T* cell suppression undoubtedly creates perspectives for the combination of immunotherapy and anti-platelet agents as a therapeutic strategy against cancer [44, 45]. The effectiveness of such therapy has been proven recently by Rachidi et al. [45] in mouse models.

### Platelets in tumor angiogenesis

Neoplastic tumors, bigger than 2 mm in diameter, need nutrients and oxygen, as well as suitable cytokines, chemokines or enzymes to grow and metastasize. Platelets are implicated in the early stages of angiogenesis e.g. in the stabilization of newly formed vessels [46]. They stimulate the activation of endothelial cells (ECs) and induce angiogenesis in vivo. Moreover, platelets store in alpha-granules several molecules which exhibit proangiogenic properties like: VEGF (vascular endothelial growth factor), PDGF (platelet derived growth factor), bFGF (basic fibroblast growth factor), EGF (epidermal growth factor) [47]. It is suggested that blood platelets can selectively release VEGF via activation of PAR-1 receptors, and that at the same time the expression of endostatin inhibiting angiogenesis is decreased [48]. The stimulation of the PAR-4 receptor triggers the completely opposite process, i.e. endostatin secretion [49]. It is believed that over 80% of circulating VEGF originates from blood platelets and megakaryocytes, which in physiological conditions promote the repair of damaged tissues and vessels, and its action is inhibited by adequate inhibitors [50].

In many cancers (e.g. lung, breast, colon, and kidney) the platelet count was found to significantly correlate with plasma or serum VEGF concentration [51–53]. High VEGF level is associated with shorter overall survival in patients with carcinoma [54, 55]. As shown in our earlier report, blood platelets form complexes with tumor cells in the blood stream. At the site of tumor cell adhesion to ECs, platelets can release their α-granule content, e.g. VEGF, which induces permeability of ECs, facilitates extravasation of cancer cells, and stimulates new vessel formation at the sites of distant metastases. The action of VEGF in neoplastic disease leads to enhanced TF expression by endothelial cells and increased release of vWF. In consequence, platelets adhere to the wall of neoplastic vessels. Moreover, abnormally developed vessels within tumors cause turbulent blood flow, which in turn is associated with the activation and adhesion of platelets and their degranulation [39]. Blood platelets can also stimulate proliferation of endothelial cells and affect the integrity of newly formed or inflamed endothelial cells. This is associated with hemorrhage prevention by releasing substances that affect the adhesion properties of cells [46]. In the course of chronic inflammation and in neoplastic disease, metalloproteinases, serine proteases and other substances that damage the vascular basement membrane are released, leading to hemorrhages. In this case, the role of blood platelets is associated with modulation of the activity of inflammatory cells or inhibition of certain substances by the secretion of such inhibitors as TIMP-1 or serpin [56].

Moreover blood platelets also synthesize and secrete a variety of proteolytic enzymes, such as metalloproteinases (MMP-1,-2, -3, -9 and -14) that degrade the vascular basement membrane and cellular matrix of tissues, which may play a role in the process of extravasation, i.e. migration of cancer cells to the extravascular space [57]. This effect can be enhanced by activation and accumulation of inflammatory cells. MMPs can regulate sCD40L shedding from platelets and pulmonary recruitment of neutrophils [58].

Enhanced proliferation and survival of transformed cells is induced by thrombin, which in turn enhances VEGF expression, increases the adhesion properties of cells and promotes formation of cancer-platelet aggregates [59]. Through the activation of the PAR-1 receptor, thrombin stimulates cancer growth and metastases [60]. Thrombin is released by active blood platelets but it can also be produced by cancer cells.

### Platelets in cancer metastasis

Metastasis formation is a most important stage of tumor progression. Transformed cells leave the tumor microenvironment and migrate in the blood to even the most distant organs and their survival before extravasation is a crucial step in metastasis [34]. Therefore, cancer cells activate blood platelets by stimulating various mediators such as cathepsin G or thrombin and by constant expression of tissue factor on their surface. The activated platelets quickly bind to the surface of cancer cells and create their coating [61]. TCIPA is considered crucial for cancer dissemination and resembles the formation of the platelet-monocytes complexes (MPAs). Thus, it can be said that TCIPA increases survival of circulating tumor cells due to the presence of integrins and the GP IIb/IIIa receptor on the platelet surface, which via fibrinogen or vWF interact with tumor integrins. The interaction of platelets with cancer cells is also mediated by P-selectin, which binds to appropriate ligands, e.g. CD24 and CD44 on cancers [62]. Platelet-covered tumor cells can migrate freely, avoiding the immune response of the host, and above all the uptake and damage by NK cells [63]. So it can indicate that platelets may provide some physical barrier to NK cells contact.

Some antiplatelet drugs can have an antimetastatic effect by TCIPA inhibition, e.g. ADP-scavenging agents, apyrase and creatine phosphokinase and also GP IIb/IIA inhibitors [64].

An experimental study, in several mouse models, indicate that antibody-induced or genetic depletion of platelet inhibits metastasis, whereas platelet reconstitution restores metastatic activity [65–67]. What’s more, platelets arrest cancer cells in capillaries at the vascular wall via P-selectin and its ligand and facilitate tumor cell extravasation to the subendothelial matrix of the distant organs by activation of the endothelial P2Y2 receptor [68, 69]. Platelet dense granules secrete ATP, which in turn bind to activated endothelial receptors. It leads to the opening of the endothelial barrier and tumor cells can transmigrate and extravasate to form metastasis loci [70], and provide a survival signal, which in turn allows a chemotactic gradient of CCL2 chemokine, which is crucial for monocytes recruitment [71, 72]. The monocytes then differentiate into metastasis-associated macrophages (MAMs) that promote extravasation [73]. Platelets together with leukocytes and tumor cells, induced another chemokine—CCL5/RANTES from EC in an experimental model of colorectal cancer, which enhanced metastatic seeding due to recruitment of monocytes [74].

Besides VEGF, platelets release growth factors to the tumor microenvironment, like TGF-β1 and PDGF, enhancing tumor metastatic potential [38] and instigate tumor cell proliferation, vessel formation and invasiveness [27, 68]. TGF-β1 derived from platelets diminishes NK granule mobilization, cytotoxicity and INF-*γ* secretion [75] and induced an EMT (epithelial to mesenchymal transition) in carcinoma cells [68] TGF-β1 activity is associated with cancer stages, and microenvironment, and can have the opposite effect. In early stages, it can act as a tumor suppressor and potentially inhibit cancer cell proliferation and tumor growth [76, 77]. However, TGF-β1 released by platelets into the microenvironment can support tumor growth and metastasis formation [76]. In many cancers inhibition of TGF-β signaling in cancer cells strongly reduced intravasation and metastasis [78]. PDGF is involved in the regulation of proteolytic enzyme activity and its elevated concentration in breast cancer reflects a more aggressive and advanced tumor phenotype [79]. Its expression has been identified in various types of tumors, e.g. prostate [66] and colorectal cancer in which it has been proposed as a prognostic marker [80].

As shown in the latest reports, blood platelets are also involved in the formation of the tumor microenvironment during metastasizing [81]. It is believed that the platelet-tumor aggregates are already responsible for neutrophil recruitment within the first two hours after cancer cells have colonized a new site. In this early phase of metastatic formation, no inflow of monocytes, lymphocytes, dendritic cells or NK cells is observed. It appears that the attraction of neutrophils to the tumor microenvironment depends on platelet activation. When functioning of these cells is impaired or in thrombocytopenia this process does not occur. The chemokine CXCL5/7 which is released from blood platelets and then binds to CXCR2 on the surface of neutrophils, is responsible for the activation and migration of granulocytes [82].

It seems that the ability of platelets to form aggregates between tumor cells, platelets and leukocytes may be a crucial step in determining tumor cell survival within the microvasculature of the target organs of metastasis [71].

### Platelets count in malignancy

The development of neoplastic disease affects not only platelet activation but also their morphological parameters, i.e. platelet count (PLT) and mean platelet volume (MPV) [83]. A significant drop in PLT—thrombocytopenia—can be associated with intensive chemotherapy and the presence of metastases in bone marrow exerting a suppressive effect on the precursor cells. Cancer patients show increased platelet turnover [84] which improves after anticancer treatment [85, 86].

A systematic drop in platelet count leads to slight bleeding from tiny vessels, whereas considerable thrombocytopenia triggers massive hemorrhages from large blood vessels [81]. Thrombocytopenia experimentally induced by a variety of mechanisms is associated with a reduction in the number of cancer metastases [87].

During the course of cancer, reactive thrombocytosis (more than 400 × 109/L) is much more common and observed in 10–57% of patients, depending on the tumor type [39]. It has long been known that the increased platelet count is associated with more advanced neoplastic disease and is an unfavorable prognostic factor in various cancers [71, 43]. Thrombocytosis is also positively correlated with shortened survival and poor prognosis [88]. However, the underlying mechanisms of thrombocytosis are not completely clear. It is suggested that enhanced production of blood platelets is stimulated by cytokines IL-1, IL-6, and growth factors: GM-CSF and G-CSF released from leukocytes during inflammation and by cancer cells themselves [89]. It is well documented that several tumor cells can produce TPO (thrombopoietin), a main cytokine directly and indirectly regulating megakaryopoiesis and thrombocytopoiesis [90]. These factors stimulate thrombocytopoiesis through binding to the receptors present on megakaryocytes Mpl (TPO-R) [77]. They act synergistically with TPO. In turn, the action of IL-6 is associated with enhanced production of TPO in the liver and a direct effect on megakaryocytes via the membrane receptor IL-6R. This means that platelet count may be markedly increased in the course of inflammation and neoplastic disease. It should also be mentioned that megakaryocytes are able to produce proinflammatory cytokines, e.g. IL-1, -3, -6, GM-CSF, stimulating endothelial cells of bone marrow vessels to produce factors that maintain maturation and differentiation of precursor cells and in consequence platelet production [91]. Moreover, it is known that bone marrow endothelial cells (BMECs) can support megakaryocytopoiesis. BMECs support proliferation and differentiation of megakaryocytic progenitor cells in vitro as well as facilitating the growth and maturation of megakaryocytes in vivo, and release some cytokines, such as IL-6 and TPO [77].

The increased platelet count in the course of neoplastic disease is detrimental and favors the risk of thrombosis which can be the first manifestation of neoplastic disease [92]. Hypercoagulability and excessive thrombin generation, which are commonly observed in the course of malignancy, may contribute to thrombosis in cancer patients, which in fact can be the first symptoms among cancer patients [93].

### Targeting platelets in cancer treatment

A wealth of data in the available literature provides information that targeting platelets in cancer treatment would be beneficial [94–98]. Only single studies indicate contrary properties to such an approach [99, 100]. The multitude of reports on this topic highlights the fact that this issue could benefit from being subjected to a separate review. However, Table 1, specially prepared for the purposes of this review, summarizes the main targets of antiplatelet therapy, their best known inhibitors, along with their mechanisms of action and the effect they have on cancer (Table 1). In the clinic, the targeting of only cyclooxygenase molecules is well-characterized [94–96, 101–103]. Studies concerning other platelet targets (P2Y12, glycoproteins, integrins, L-, P-selectins, PAR-1,-4, thrombin activity, podoplanin-CLEC-2) have so far been utilized mainly on cell lines or animal models [64, 104–109]. The targeting of cancer cell-platelet interaction reduces both metastasis and thrombosis [97, 110–113]. However, this interaction is still not completely understood, thus future studies are required to explicitly explain the role of anti-platelet therapy in cancer prevention and treatment.

**Table 1:** Targeting platelets in cancer treatment

Target	Inhibitors	Mechanism of action	Effect in cancer treatment
COX-1 COX-2	Aspirin Other COX inhibitors: celecoxib, rofecoxib, valdecoxib, parecoxib, etoricoxib	Inhibition of thromboxane and prostaglandin generation, which prevents platelet activation, aggregation, degranulation and TCIPA [110, 111] Inhibition of platelet proangiogenic activity (release of VEGF and endostatin) [47] Induction of platelet apoptosis via caspase-3 activation [114] Platelet-mediated inhibition of oncoprotein c-MYC proto-oncogene expression in cancer cells [115]	Reduction of incidence of colorectal cancer [116], colorectal adenomas [102, 117], breast cancer [94], lung cancer [95], prostate cancer [96, 103] in humans Reduction of cancer growth in colorectal and pancreatic cancer in humans [115] Reduction of metastasis of breast cancer in humans [97, 113], adenocarcinoma in humans [112], colorectal cancer in mouse [118], melanoma in mice [119] Reduction of recurrence of colorectal cancer [120], colorectal adenomas [117, 121], breast cancer [122], HBV-related hepatocellular carcinoma [98] in humans Reduction of cancer-associated death in colorectal cancer, adenocarcinomas [123, 124], breast cancer [113], prostate cancer [125] in humans Improvement of treatment of breast cancer patients [97]
P2Y12	Thienopyridines Clopidogrel Ticagrelor Ticlopidine Cangrelor Prasugrel Apyrase	Inhibition of thromboxane generation and P-selectin and integrin activation, which prevents platelet activation, adhesion, aggregation and TCIPA [104, 113, 126–131] Inhibition of platelet degranulation (release of ADP, ATP, TGFβ1) [70, 105, 132–134] Inhibition of platelet proangiogenic activity (release of VEGF, endostatin) [135, 136] Inhibition of platelet shape change and diminished platelet-induced EMT-like transformation of cancer cells [105]	Reduction of metastasis of breast cancer in cells lines and mouse models [104], LLC in mice [105], melanoma in mice [132, 134, 136], breast cancer in mice [134] Decreased formation of ovarian cancer in cell lines model [137] Improvement in survival among HBV-related hepatocellular cancer patients [98] Moderate antiplatelet therapy > 2 years, or aggressive regimens including triple therapy for < 1 year, the risks of solid cancer emerge [100]
PAR-1, PAR-4	RWJ58259 SCH-79797 RWJ-56110 SCH 19,197 siRNA incorporated into neutral liposomes (DOPC) Kallikrein	Inhibition of TCIPA [106, 138, 139] Inhibition of platelet proangiogenic activity (release of VEGF, endostatin, IL-8) [106, 140]	Inhibition of growth, weight, and metastasis of lung cancer in mice [106] Reduction of melanoma metastasis in mice[65, 106, 141]
GLYCOPROTEINS	GP IIb/IIIa inhibitors (abciximab, eptifibatide, tirofiban, triflavin, rhodostomin, trigramin, XV454, 7E3 F(ab')(2)) GPIIIa49-66 inhibitors (singlechain antibody/scFv Ab; A11) GP1b/IX/V inhibitors GPVI inhibitors	Inhibition of the final pathway of platelet aggregation and thus reduction of TCIPA [67, 109, 142–144] Inhibition of platelet proangiogenic activity (release of VEGF) [145]	Reduction of metastasis of liver cancer in cell line models [109], LLC in mice [142, 144] Increased metastasis of melanoma in animal models [99]
INTEGRINS, L-, P-selectins	Rhodostomin Holothurian glycosaminoglycan (hGAG) Heparin, dermatan sulfates	Inhibition of TCIPA [146–148]	Reduction of metastasis of melanoma in mice [64, 148] Improvement in survival in mouse colon carcinoma metastatic model [149]
THROMBIN ACTIVITY	Hirudin Apyrase Heparins Dansylarginine N-(3-ethyl-1,5-pentanediyl) amide Bivalirudin Lepirudin Desirudin Argatroban Reversible thrombostatin FM19	Inhibition of TCIPA [150, 151] Inhibition of platelet proangiogenic activity (release of VEGF, expression of Twist and GRO-α) [152–154]	Reduction of metastasis of melanoma in mice [107, 155]; LLC in mice [151]; prostate cancer in mice [156]; breast cancer in mice [157]; human laryngeal cancer in cell line model [146] Reduction of growth of ovarian cancer in humans [158]; prostate cancer in mice [156]; humans laryngeal cancer in cell line model [146]
PODOPLANIN-CLEC-2	Monoclonal antibody: NZ-1, SZ168 Non-cytotoxic 5-nitrobenzoate compound (2CP) PLAG3/4 inhibitors	Inhibition of TCIPA [34, 36, 159, 160]	Reduction of metastasis of melanoma in humans [108], C6/Lung cells in mice [159] Improvement of treatment with cisplatin in the mouse xenograft model [159] Increased survival in glioblastoma mouse-model [161]

## Conclusions

There is growing evidence for a close relationship between platelets and cancer development. This specific cross-talk between tumor cells and platelets has been noticed by many authors. Taking into the account the role of platelets in the development of cancer, we have decided to introduce a new term: tumor associated platelets—TAPs. To the best of our knowledge, so far nobody has used this term. Platelets are indirectly activated by tumor cells via coagulation activation induced mainly by TF present on tumor cells. Activated platelets protect tumor cells against cytotoxic activity of NK cells, surrounding them and forming tumor cell-platelet aggregates. Activated platelets also release a number of factors from their granules that can enhance the development of the tumor microenvironment, vascular neoangiogenesis and in consequence metastasis formation. On the other hand, it also has been indicated that factors released from platelets can induce a cytotoxic effect on the proliferating neoplastic cells, and even enhance apoptosis. In respect to the above mentioned information it should be noted that TAPs’ role seems to be more complex as compared to tumor associated neutrophils and macrophages, which does not allow for the easy division of TAPs into TAP P1 and TAP P2. Nevertheless, better exploring the interactions between platelets and tumor cells could help to propose new therapeutic goals that support or improve the effectiveness of cancer treatment.

## Electronic supplementary material

Below is the link to the electronic supplementary material.

Supplementary file1 (DOC 76 KB)

## Data Availability

The datasets generated and analyzed during the current study are not publicly available but all are kept at the Medical University of Bialystok and available from the corresponding author (VD-P) on reasonable request.
