# Supplementary material for: Inflammatory cell-associated tumors. Not only macrophages (TAMs), fibroblasts (TAFs) and neutrophils (TANs) can infiltrate the tumor microenvironment. The unique role of tumor associated platelets (TAPs)
Source: Cancer Immunol Immunother. 2020 Nov 3;70(6):1497–510. doi: 10.1007/s00262-020-02758-7 (PMC8139882; doi:10.1007/s00262-020-02758-7)
Supplement: Supplementary file 1 — Supplementary file1 (DOC 76 KB) [file 262_2020_2758_MOESM1_ESM.doc]

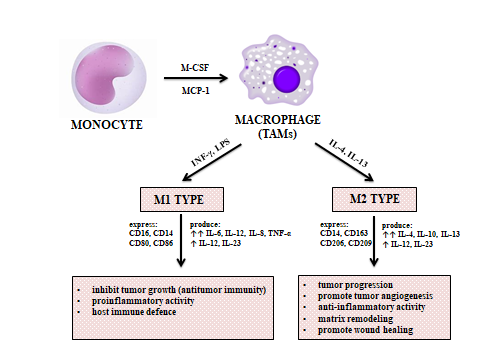


**Figure 1.** Tumor associated macrophages (TAMs).

**Legend to the Figure 1:** CD - cluster of differentiation, IL -4, -6, -8, -10, -12, -13, -23 - interleukin -4, -6, -8, -10, -12, -13, -23, INF-γ - interferon gamma, LPS - lipopolysaccharide, M-CSF - macrophage colony-stimulating factor, MCP-1/CCL2 - monocyte chemoattractant protein-1, TAMs - tumor‐associated macrophages.
